# Body-oriented interventions to promote preschoolers’ social–emotional competence: a quasi-experimental study

**DOI:** 10.3389/fpsyg.2023.1198199

**Published:** 2023-07-31

**Authors:** Andreia Dias Rodrigues, José Marmeleira, Clarinda Pomar, Elsa Lamy, Daniela Guerreiro, Guida Veiga

**Affiliations:** ^1^Departamento de Desporto e Saúde, Escola de Saúde e Desenvolvimento Humano, Universidade de Évora, Évora, Portugal; ^2^Comprehensive Health Research Centre (CHRC), Universidade de Évora, Évora, Portugal; ^3^Departamento de Pedagogia e Educação, Escola de Ciências Sociais, Universidade de Évora, Évora, Portugal; ^4^Centro de Investigação em Educação e Psicologia, Universidade de Évora, Évora, Portugal; ^5^MED - Mediterranean Institute for Agriculture, Environment and Development, Universidade de Évora, Évora, Portugal; ^6^CHANGE - Global Change and Sustainability Institute, Universidade Évora, Évora, Portugal

**Keywords:** mind–body, loose parts play, relaxation, social–emotional development, children, psychomotor intervention, saliva

## Abstract

**Introduction:**

Social–emotional competence is foundational to children’s health and well-being. Body-oriented interventions, such as relaxation or play based interventions, have been shown to promote social–emotional competence, however more studies are needed to better understand the specific benefits of each type of body-oriented approach.

**Objective:**

The present study aimed to examine the chronic and the acute effects of three body-oriented intervention programs (loose parts play, relaxation and combining loose parts play and relaxation) on preschoolers’ social–emotional competence.

**Methods:**

A quasi-experimental study was carried out, including 62 preschoolers (4.44 ± 0.93 years) that were allocated into 4 groups: Loose Parts Play program (*n* = 17); Relaxation program (*n* = 17); Combined program (*n* = 13); and Waitlist Control Group (no intervention; *n* = 15). All three intervention programs had a 12-week duration, with biweekly sessions of 30-min, implemented in the preschool outdoors. To examine the chronic effects of the intervention programs, all instruments (parents’ and preschool teacher’s questionnaires, tasks and saliva) were collected at baseline and after the 12-week period. To examine the acute effects, saliva samples were collected immediately before and after the 1st and the 24th sessions, with a total of 4 collections per child.

**Results:**

Both loose parts play and relaxation interventions significantly improved (*p* < 0.05) children’s positive emotion expression. Several within-groups changes were found for the Loose parts play, Relaxation and Combined programs.

**Conclusion:**

Body-oriented interventions effectively promote preschoolers’ social–emotional competence.

## 1. Introduction

During preschool years, children experience a significant development of social–emotional competence, which is foundational to children’s health and well-being (Jones et al., 2015; Cornell et al., 2017; Domitrovich et al., 2017). The school context has proved to be a critical environment where children develop their social–emotional competence, providing a key opportunity to observe and improve children’s abilities to interact with peers and preschool teachers as they cooperate and negotiate to complete daily tasks and resolve conflicts (Jones et al., 2015).

The outdoor is a rich context in terms of sensorial and social–emotional experiences, bringing a variety of benefits to children’s social–emotional development (Kemple et al., 2016; Tandon et al., 2018). The perceived freedom and space facilitate movement and emotional expression and regulation. Indeed, recent systematic reviews showed that natural environments reduce stress, improve mental health (Tillmann et al., 2018), and facilitate children’s social–emotional adaptive behaviors (Mygind et al., 2021; Johnstone et al., 2022).

According to the Collaborative for Academic, Social, and Emotional Learning model, social–emotional competence can be conceptualized in five core clusters: self-awareness, self-management, social awareness, relationship skills, and responsible decision-making, which have a significant role in different aspects of life (Weissberg et al., 2015; Collaborative for Academic, Social, and Emotional Learning, 2020). These five core categories encompass a constellation of other competencies, such as emotion understanding, self-regulation, communication, and problem-solving skills (Denham, 2006; Collaborative for Academic, Social, and Emotional Learning, 2013, 2020). Social–emotional competence at an early age has been positively related to health, positive interpersonal relationships, well-being, and professional success, in the short and in the long-term (Durlak et al., 2011; Jones et al., 2015; Cornell et al., 2017; Eklund et al., 2018). Hence, social–emotional competence has been recognized as a key domain that should be an integral part of early childhood education, such as academic skills (Organization for Economic Co-operation and Development, 2017; Collaborative for Academic, Social, and Emotional Learning, 2020).

Considering the recognized importance of social–emotional competence in the early years, several intervention programs have been implemented within school context aiming to promote preschoolers’ social–emotional competence. A recent systematic study (Dias Rodrigues et al., 2022a) has shown that body-oriented interventions effectively promote social–emotional competence, although more studies are needed to better understand the benefits regarding preschool age. Body-oriented interventions are based on the premise that emotional and bodily experiences are associated, aiming to promote the awareness of the body, the connection between the body and emotions, and the body in relation to others (Röhricht, 2009; Bloch-Atefi and Smith, 2014; Rosendahl et al., 2021). Body-oriented interventions encompass different types of approaches, such as play, relaxation, or dance. A recent systematic review (Dias Rodrigues et al., 2022b) revealed that the majority of body-oriented approaches implemented in preschool context are focused on play (10 in the 19 studies included in the review), and other approaches such as relaxation or combined programs (e.g., play and relaxation) are less studied. Such fact highlights the need for more studies focused on the effects of other body-oriented approaches on preschoolers’ social–emotional competence (Dias Rodrigues et al., 2022b).

Play has an important role in preschoolers’ social–emotional development, and this may explain the fact that play-based interventions are the body-oriented approaches most applied in the preschool context (Dias Rodrigues et al., 2022b). Although most studies focused on the effects of pretend and physical play, research on loose parts play is still scarce (Gibson et al., 2017; Dias Rodrigues et al., 2022b). Implementing loose parts in children’s play provide opportunities for free play, allowing children to choose and create their own playful activities, to make independent decisions, and to investigate their social world (Gibson et al., 2017), been also considered important to children’s healthy development (Milteer et al., 2012). In this way, loose parts play introduces a range of open-ended and manipulable materials into children’s play setting (e.g., stones, sticks, card boxes, clothespins), promoting their engagement (Gull et al., 2019). Research has shown the positive influences of loose parts play parts in children’s social–emotional outcomes, such as communication (Maxwell et al., 2008), negotiation skills (Maxwell et al., 2008), social interaction (Hyndman et al., 2014) and cooperation (Flannigan and Dietze, 2017), and problem solving (Flannigan and Dietze, 2017). Despite this, the need for more studies focusing on the effects of loose parts play on preschoolers’ social–emotional competence is evident (Gibson et al., 2017; Dias Rodrigues et al., 2022b).

Relaxation is another type of body-oriented intervention that has been implemented in the preschool context. Relaxation intervention programs encompass different techniques aiming to obtain the relaxation response, either through body functions regulation (e.g., breath, tonus) such as breathing techniques or muscular relaxation, and/or through cognitive regulation (e.g., attention) such as mindfulness (Veiga and Marmeleira, 2018). Regardless the technique used, the activities must be adapted to the physical and emotional needs of children (Cooke et al., 2021). Through relaxation children are able to enhance their awareness and regulation of their bodies, emotions, and thoughts (Veiga and Marmeleira, 2018; Cunsolo et al., 2021), as well to face negative stress inductors situations, and look for solutions to better deal with problems (Cunsolo et al., 2021). Despite the lack of studies, in the last few years a growing body of evidence supports the effectiveness of relaxation programs in the educational context, showing the positive influences of relaxation on preschoolers’ social–emotional competence, such as emotion regulation (Flook et al., 2015), social competence (Flook et al., 2015; Marmeleira et al., 2018), and behavior problems (Moreno-Gómez and Cejudo, 2018).

Researchers sometimes combine the use of different approaches to gain the strengths of two or more approaches. Hence, there are potential benefits of combining loose parts play and relaxation for preschoolers’ social–emotional competence. For example, in Lee et al. (2020), a 1-week program with daily sessions of 70 min that combined loose parts play and mindfulness activities, showed positive effects in almost all the outcomes studied, such as happiness after play, play disruption, play disconnection, play interaction, play intensity, and play skill.

The main purpose of this study was to examine the impact of body-oriented intervention programs on preschoolers’ social–emotional competence. More specifically, this study aimed to investigate the chronic and acute effects of three different body-oriented programs, namely Loose parts play, Relaxation, and Combined loose parts play and relaxation program, on preschoolers’ social–emotional outcomes. These outcomes include emotion discrimination, identification, and recognition; positive and negative emotion expression; self-regulation; stress regulation; social competence; externalizing problems; and conflict resolution abilities.

It was hypothesized that preschoolers’ social–emotional competence (emotion discrimination, identification, and recognition; positive and negative emotion expression; self-regulation; stress regulation; social competence; externalizing problems; and conflict resolution abilities) would benefit from 12 weeks of the three body-oriented intervention programs and that the programs would produce chronic and acute effects on preschoolers’ social–emotional competence.

## 2. Materials and methods

### 2.1. Participants

Children were recruited from schools with preschool education, in Évora, Portugal, where the study was carried out. Parents provided written informed consent for participation, and children provided verbal consent. The inclusion criteria were (a) participants’ age between 3 and 6 years, (b) do not have participated in a similar intervention program within the last 6 months, and (c) do not have a physical condition that can affect the participation in the program. Sixty-nine informed consents were given to children’s parents. Of these, 65 signed informed consents were returned, approving the child’s participation in the study. Three of the participants left the kindergarten during the intervention period, and therefore were not included in the study.

Sixty-two preschool aged children participated in the study, being allocated by convenience (i.e., each classroom represented one group) to the Loose Parts Play Group (LPPG, *n* = 17), the Relaxation Group (RG, *n* = 17), the Combined Loose Parts Play and Relaxation Group (CG, *n* = 13), and to the Waitlist Control Group (WCG, *n* = 15).

Table 1 shows the main descriptive characteristics of the participants. There were no significant differences between groups regarding age and sex. All the participants lived in the city of Évora. The majority of the participants (47%, *n* = 29) had one sibling, and 35% (*n* = 22) were only child. The remaining participants had 2 siblings (15%, *n* = 9), or 3 (3%, *n* = 2). More than half of the children (56%, *n* = 35) had their own bedroom, and 85% (*n* = 53) had an outdoor public space in the vicinity of their home where they weekly went to play.

**Table 1:** Socio-demographic characteristics of the participants.

	LPPG (*n* = 17)	RG (*n* = 17)	CG (*n* = 13)	WCG (*n* = 15)	Total (*N* = 62)
Age (years)	4.41 ± 1.28	4.35 ± 0.79	4.14 ± 0.69	4.80 ± 0.77	4.44 ± 0.93
Girls (%)	35	53	69	60	53
Boys (%)	65	47	31	40	47

Note: LPPG, loose parts play group; RG, relaxation group; CG, combined group; WCG, waitlist control group.

### 2.2. Procedures

This study was approved by the Ethics Committee of the University of Évora and was carried out under the standards set by the Declaration of Helsinki (General Assembly of the World Medical Association, 2014). All the collected data was fully encrypted to ensure the privacy of the participants.

To examine the chronic effects, data were collected at baseline and at the end of the 12-week period (post-intervention). The tasks were individually applied and presented as games to the children in a quiet room of the kindergarten (10–15 min). Questionnaires were also delivered to parents and preschool teachers.

To measure the acute effects of the intervention programs, salivary cortisol was also measured at the beginning and end of the 1st and the last (24th) sessions. Preschool teachers were asked to restrict children potential cortisol-altering substances 1 h before the testing sessions, such as food and vigorous physical exercise.

### 2.3. Outcomes and measures

#### 2.3.1. Emotional competence

Emotion understanding encompasses the abilities of discrimination, identification, and recognition of emotions (Wiefferink et al., 2013; Rieffe and Wiefferink, 2017). In this way, emotion discrimination was measured through the Emotion-discrimination Task, following Wiefferink et al. (2013; Veiga et al., 2017) protocol. First, two non-emotional sorting tasks (flowers versus cars; heads with hats versus heads with glasses) were applied to reassure the child’s ability to sort cards. After completing this control task, the child was asked twice to place six cards, within two possible categories. At first, the cards involved happy versus unhappy faces, and secondly angry versus sad faces were presented. For each card was placed correctly, one point was counted, and scores were averaged, with a minimum score of 0 and a maximum score of 3 points.

Emotion identification was measured through the Emotion-identification Task, also following the protocol of Wiefferink et al. (2013); Veiga et al. (2017). In this task, the child had to point to the facial expressions according to the emotion words (happiness, sadness, fear, and anger) instructed by the experimenter (i.e., “Who looks happy? Who looks sad?”). For each facial emotional expressions that were correctly identified 1 point was counted. Scores were averaged to reflect a total score from 0 to 2.

Emotion recognition was measured through the subscale Others’ Emotion Recognition (6 items) of the Portuguese version of the Emotion Expression Questionnaire (EEQ; Rieffe et al., 2010; Veiga et al., 2017). This subscale is comprised by 6 items (e.g., “Does your child know when you are angry?,” “Does your child know when you are happy?”) scored in a 5-point scale [0 = (almost) never, 1 = rarely, 2 = sometimes, 3 = often, 4 = (almost) always], rated by parents regarding the extent to which the child can recognize parents’ or others’ emotions. Scores were averaged to reflect a total score from 0 to 4. The reliability of this subscale was acceptable with Cronbach’s alpha value of 0.75, and also the inter-item correlation value (0.36).

Positive emotion expression, and negative emotion expression was measured through the Positive Emotion Expression subscale (6 items) and Negative Emotion Expression subscale (8 items) from the EEQ (Rieffe et al., 2010; Veiga et al., 2017). Parents scored on a 5-point scale [0 = (almost) never, 1 = rarely, 2 = sometimes, 3 = often, 4 = (almost) always], the frequency, intensity, and duration of child’s expressions of positive emotions such as happiness or joy (e.g., “How often does your child experience joy?,” “How happy is your child?”), and negative emotions such as anger or sadness (e.g., “How often does your child experience fear?,” “How angry is your child?”). Scores were averaged. The reliability of positive and negative emotion expression subscales was acceptable and good, respectively, with Cronbach’s alpha value of 0.71 and 0.81. The inter-item correlation values were acceptable (0.20 and 0.34, respectively).

Self-regulation was measured through the Portuguese version of Head-Toes-Knees-Shoulders task (HTKS; Ponitz et al., 2009; Cadima et al., 2015). HTKS has three sections with up to four paired behavioral rules: “touch your head” and “touch your toes”; “touch your shoulders” and “touch your knees.” In the first section, the child is instructed to touch her head and toes in an opposite manner from what she is instructed (e.g., when the child is asked to touch the feet, he/she should touch the head). The child is asked to keep doing the opposite of the interviewer command throughout the test. In the second section, knees and shoulders are added. In the third section, the rules are switched so that head and knees go together, and shoulders and toes go together. Each section includes 10 trials, and for each trial, the child received a score of 0 (incorrect), 1 (self-correct), or 2 (correct). Scores were summed to reflect a total score from 0 to 60.

Stress regulation was measured through salivary cortisol levels (μg/dl). Saliva samples were collected at the same time and in the same place where the interventions occurred, before and after the 1st and 24th sessions, with a total of 4 collections per child. Samples were collected directly from each child’s mouth, without stimulation, by passive droll during 5 min to a polyethylene tube. Then, the tube was maintained on ice and further kept at −20°C, until laboratory analysis. For the analyses, samples were thawed and then centrifuged for 20 min at 13000 g, 4°C, for removal of food debris, mucins, and cells. After this process, cortisol determination was performed using the Salimetrics® Cortisol Enzyme Immunoassay (EIA) Kit, following manufacturer instructions and absorbance reading was carried out at 450 nm, in a microplate reader (Glomax, Promega).

#### 2.3.2. Social competence

Social competence was obtained through the Portuguese version of the Strengths and Difficulties Questionnaire (SDQ; Goodman, 1997; Muris et al., 2003), combining the prosocial behaviors scale (5 items), and the positive items of the peer problems scale (2 items) (Veiga et al., 2017). The SDQ was administered to preschool teachers and parents, who rated on a 3-point scale (0 = not true, 1 = somewhat true, and 2 = certainly true) the degree to which each item represented the child’s behavior in the last 3 months. Scores were averaged to reflect a total score from 0 to 2. This scale showed good and acceptable reliability, with a Cronbach’s alpha of 0.84 for preschool teachers’ and of 0.73 for parents’ measurements. The inter-item correlations values for both questionnaires were good (0.45 and 0.29, respectively).

Externalizing problems were also obtained through the procedure of Veiga et al. (2017), combining the behavior problems scale (5 items), and hyperactivity scale (5 items) of the Strengths and Difficulties Questionnaire (SDQ; Goodman, 1997; Muris et al., 2003). Parents rated on a 3-point scale (0 = not true, 1 = somewhat true, and 2 = certainly true) the degree to which each item represented the child’s behavior in the last 3 months. Scores were summed to reflect a total score from 0 to 2. This scale showed acceptable reliability with Cronbach’s alpha value of 0.71, and an inter-item correlation value also acceptable (0.18).

Conflicts resolution was measured through the conflict resolution strategies subscale (6 items) of the Portuguese version of Social Strategies Rating Scale (SSRS; Beckman and Lieber, 1994; Fialho and Aguiar, 2017). The SSRS was administered to preschool teachers, who rated on a 5-point scale (1 = never, 3 = half of the time, 5 = almost always) how often the child uses social strategies during interactions with peers on preschool context. Scores were summed to reflect a total score from 1 to 5. This scale showed good reliability with a Cronbach’s alpha value of 0.83, and a good inter-item correlation (0.43).

### 2.4. Intervention programs

All the intervention programs (loose parts play, relaxation, combined) were implemented by a psychomotor therapist in the school playground (except for the days when it rained, being implemented in the classroom) involving the whole class. All sessions had a 30-min and were carried out twice a week for 12 weeks.

The Loose parts play program sessions began with an initial dialogue (2 min), a main section (25 min), and a final ritual (3 min). During the main section, participants played freely with any loose parts available in the playground. At the beginning of the program, smaller loose parts (e.g., card boxes of small sizes, bottle caps of different sizes and colors, sticks, stones, fabric, bottles, clothes springs) were introduced, affording (mostly solitary) object exploration, and therefore stimulating children’s curiosity and awareness of their bodies in action. As the sessions progressed, bigger loose parts (e.g., larger card boxes, tires, tape, strings, demijohns, tubes) were added. As the exploration and use of bigger materials require more children, collaborative and social behaviors were therefore afforded. Nevertheless, it is important to note that despite the different loose parts, children had total freedom to choose, decide and structure their own play.

The Relaxation program was structured with an initial dialogue (2 min), a main section (25 min), and a final ritual (3 min). During the main section, relaxation activities focused on Jacques Choque Method (Choque, 1994) were implemented, being adapted to the interests of the intervention group (e.g., using favorite fictional characters or animals, music, or other activities that can be adapted to the goals of the programs, such as “Captain’s Orders”). An example of one activity was the “Balloon belly breathing,” where children had to imagine that they belly was a balloon, and while they breath in the balloon gets bigger, and when they breath out the balloon empties. All the children were actively engaged in all the activities. The goals of the intervention program were organized in four cumulative stages: (a) promotion of body and emotion awareness – 1st to 6th sessions; (b) promotion of self-regulation – 7th to 12th sessions; (c) promotion of the awareness of others’ emotions (13th to 18th sessions); and (d) promotion of the body in relation with others and problem solving abilities, which means helping in the creation and maintenance of positive interpersonal relationships (19th to 24th sessions). As the program progressed, activities complexity was increased (e.g., at the beginning, stretching activities were focused on a smaller number of muscle groups, but as the sessions progressed, a greater number of muscle groups were combined), as well the need of cooperation with peers. In particular, while at the beginning of the program the activities were individually performed, as the sessions progressed, activities gradually involved more peers (from pairs to the whole group).

The Combined program was structured with an initial dialogue (3 min), a loose parts play moment (15 min), a relaxation moment (10 min), and a final ritual (2 min). The loose parts play moment followed the same principles describe above regarding the Loose parts play intervention program, as well as the moment of relaxation, which was comprised by 2 relaxation activities per session.

All sessions were planned and conducted by a psychomotor therapist and the preschool teacher was available to children during the intervention sessions.

The WCG participants maintained their usual daily live activities during the intervention period.

### 2.5. Statistical analyses

Psychometric properties of the questionnaires used to assess emotion recognition, positive and negative emotion expression, social competence, externalizing problems, and conflict resolution were examined through Cronbach’s alpha coefficient and item-total correlation.

A descriptive analysis (frequencies, means and SD) and normality tests (Shapiro–Wilk test) were performed for all the variables. As the majority of the variables did not have a normal distribution, non-parametric Kruskal–Wallis *H* tests (one-way ANOVA on ranks) with a *post hoc* test (pairwise comparisons) were carried out to compare between group changes after the intervention program. Significance levels were adjusted using the Bonferroni correction for multiple comparisons. Scores were tested for change over time using *post hoc* pairwise comparisons with the Mann–Whitney test with a Bonferroni adjustment applied to compensate for the multiple comparisons between four groups, and significance was set at *p* < 0.0083 (0.05/6 comparisons = 0.0083). Changes within groups were examined using the Wilcoxon Signed test, and significance was set at *p* < 0.05. The results are expressed as median and interquartile range (IQR).

Analyses were conducted with the statistical software Statistical Package for the Social Sciences (Version 27.0; IBM SPSS Inc.).

## 3. Results

All schools contacted to implement the programs accepted to participate in the study. All intervention programs had good compliance, exceeding 83% (20 in 24 sessions) in the Loose parts play program, 71% (17 in 24 sessions) in the Relaxation program, and 79% (19 in 24 sessions) in the Combined program. Children’s absence was related to school absence on the days those sessions occurred. Although children were informed that they were free to not participate in the sessions, that never happened.

### 3.1. Emotional competence

As shown in Table 2 the groups did not show statistically significant differences in the dependent outcomes at baseline. Kruskal–Wallis test showed that median emotion discrimination and emotion identification scores were not statistically significantly different between the groups. Statistically significant increases were found in emotion discrimination scores from baseline to 12 weeks in the LPPG (*ΔMdn* = 0.75, *z* = −2.82, *p* = 0.005), RG (*ΔMdn* = 0.75, *z* = −2.80, *p* = 0.005), in CG (*ΔMdn* = 1.00, *z* = −2.95, *p* = 0.003) and in the WCG (*ΔMdn* = 0.25, *z* = −2.04, *p* = 0.041).

**Table 2:** Scores on the emotion discrimination, emotion identification, emotion recognition, positive emotion expression, negative emotion expression, and behavioral self-regulation at baseline and at 12 weeks.

Emotional competence (Min-Max)	Group	Baseline *Mdn* (IQR)	12 weeks *Mdn* (IQR)	Kruskal–Wallis test value of *p*	*Post hoc* test^‡^
Emotion discrimination (0–3)	LPPG	2.00 (1.12)	2.75 (0.75)**	0.31	
	RG	2.00 (1.00)	2.75 (1.00)**		
	CG	1.75 (1.13)	2.75 (0.50)**		
	WCG	1.25 (1.00)	2.00 (0.50)*		
Emotion identification (0–2)	LPPG	1.75 (1.13)	2.00 (0.00)**	0.25	
	RG	1.50 (1.13)	2.00 (0.00)**		
	CG	1.75 (0.75)	2.00 (0.00)*		
	WCG	1.50 (0.50)	1.75 (0.75)		
Emotion recognition (0–4)	LPPG	2.83 (1.34)	2.83 (1.25)	0.04^†^	
	RG	2.67 (1.00)	2.83 (0.67)*		
	CG	2.83 (0.50)	2.83 (1.08)		
	WCG	2.83 (0.67)	3.00 (0.67)		
Positive emotion expression (0–4)	LPPG	3.33 (0.58)	3.50 (0.25)*	<0.001	LPPG > CG
	RG	3.17 (0.58)	3.67 (0.17)*		RG > CG
	CG	3.33 (0.58)	3.33 (0.58)		
	WCG	3.50 (0.50)	3.17 (0.50)*		
Negative emotion expression (0–4)	LPPG	1.28 (1.21)	1.28 (0.78)	0.34	
	RG	1.86 (1.07)	1.86 (0.57)		
	CG	1.14 (0.36)	1.28 (0.36)		
	WCG	1.86 (0.86)	1.28 (0.56)		
Self-regulation (0–60)	LPPG	4.00 (16.50)	19.00 (34.00)**	0.28	
	RG	1.00 (6.50)	13.00 (28.50)**		
	CG	2.00 (4.00)	19.00 (31.00)**		
	WCG	4.00 (12.00)	2.00 (24.00)		

Note: Data are expressed as median and interquartile range (IQR). *p < 0.05, **p ≤ 0.01, changes within the group using the Wilcoxon test. ^‡^Pairwise comparisons of change scores using Mann–Whitney test. ^†^Kruskal–Wallis H test statistically significant, but no statistically significant pairwise comparisons were found. LPPG, loose parts play group; RG, relaxation group; CG, combined group; WCG, waitlist control group.

Statistically significant increases were found in emotion identification scores from baseline to 12 weeks in the LPPG (*ΔMdn* = 0.25, *z* = −3.09, *p* = 0.002), RG (*ΔMdn* = 0.25, *z* = −2.89, *p* = 0.004), and in the CG (*ΔMdn* = 0.25, *z* = −2.38, *p* = 0.018).

Kruskal–Wallis test showed that median emotion recognition scores were statistically significantly different between the different groups, *χ2*(3) = 8.038, *p* = 0.045. Subsequently, *post hoc* test did not reveal significant differences between groups. Despite this, within group analyses revealed a statistically significant increase in RG emotion recognition scores from baseline to 12 weeks (*ΔMdn* = 0.33, *z* = −2.10, *p* = 0.035).

Positive emotion expression scores were statistically significantly different between the different groups, *χ2*(3) = 17.563, *p* < 0.001. *Post hoc* analysis revealed statistically significant differences in this outcome between the LPPG (*ΔMdn* = 0.17) and the WCG scores (*ΔMdn* = −0.17) (*p* = 0.006), and the RG (*ΔMdn* = 0.33) and the WCG scores (*ΔMdn* = −0.17) (*p* = 0.002). Within group analysis showed that statistically significant increases were observed from baseline to 12 weeks in the LPPG (*ΔMdn* = 0.17, *z* = −2.10, *p* = 0.035), and in the RG (*ΔMdn* = 0.33, *z* = −2.16, *p* = 0.031). We also observed a statistically significant decrease from baseline to 12 weeks in the WCG (*ΔMdn* = −0.17, *z* = −2.41, *p* = 0.016).

In self-regulation no statistically significant differences were observed between groups, but Wilcoxon signed-rank test revealed that self-regulation scores significantly increased from baseline to 12 weeks in the LPPG (*ΔMdn* = 6.00, *z* = −2.73, *p* = 0.006), RG (*ΔMdn* = 12.00, *z* = −2.82, *p* = 0.005), and in the CG (*ΔMdn* = 9.00, *z* = −2.67, *p* = 0.008).

### 3.2. Social competence

As shown in Table 3, at baseline, the groups did not show statistical differences in most of the dependent outcomes. The exceptions were: the outcome ‘social competence’ reported by preschool teachers, in which the LPPG had 0.58 more points than the CG, and the WCG had 0.58 more points than the CG; and ‘conflict resolution’, in which the WCG had 1.34 more points than the RG.

**Table 3:** Scores on social competence, externalizing problems, and conflict resolution at baseline and at 12 weeks.

Social competence (Min–Max)	Group	Baseline *Mdn* (IQR)	1 weeks *Mdn* (IQR)	Kruskal–Wallis test value of *p*	*Post hoc* test^‡^
Social competence (PT) (0–2)	LPPG	1.86 (0.29)^a^	1.43 (0.57)**	0	RG > LPPG
	RG	1.71 (0.58)	1.86 (0.50)		
	CG	1.28 (0.71)^a,b^	1.14 (0.86)		
	WCG	1.86 (0.20)^b^	1.57 (0.58)**		
Social competence (P) (0–2)	LPPG	1.71 (0.50)	1.86 (0.29)	0.21	
	RG	1.71 (0.43)	2.00 (0.22)**		
	CG	1.86 (0.50)	1.86 (0.36)		
	WCG	1.71 (0.43)	1.86 (0.57)		
Externalizing problems (P) (0–2)	LPPG	0.90 (0.55)	1.86 (0.29)	0.24	
	RG	0.80 (0.65)	2.00 (0.22)		
	CG	0.50 (0.60)	1.86 (0.35)		
	WCG	0.80 (0.10)	1.86 (0.58)*		
Conflict resolution (PT) (1–5)	LPPG	3.50 (1.50)	3.17 (1.58)	0.01^†^	
	RG	2.83 (0.83)^a^	3.50 (1.50)		
	CG	3.17 (1.08)	3.67 (1.17)		
	WCG	4.17 (1.17)^a^	3.17 (1.00)**		

Note: Data are expressed as median and interquartile range (IQR). **p ≤ 0.01changes within the group using the Wilcoxon test. ^‡^Pairwise comparisons of change scores using Mann–Whitney test (*p* < 0.05). ^a,b^ *p* < 0.05 comparison between groups at baseline through Kruskal–Wallis *H* test. PT, preschool teachers; P, parents; LPPG, loose parts play group; RG, relaxation group; CG, combined group; WCG, waitlist control group.

Social competence scores reported by preschool teachers were statistically significantly different between the different groups, *χ2*(3) = 14.001, *p* = 0.003. *Post hoc* analysis revealed statistically significant differences in this outcome between the LPPG (*ΔMdn* = −0.43) and the RG scores (*ΔMdn* = 0.00) (*p* = 0.002). Within group analysis showed that statistically significant decreases were observed from baseline to 12 weeks in the LPPG (*ΔMdn* = −0.43, *z* = −3.05, *p* = 0.002), and in the WCG (*ΔMdn* = −0.14, *z* = −2.60, *p* = 0.010).

Statistically significantly differences were found between groups in conflict resolution, *χ2*(3) = 12.360, *p* = 0.006. Despite *post hoc* analysis did not reveal between each group occurred this significant difference, within group analysis revealed a significant decrease of conflict resolution from baseline to 12 weeks in the WCG (*ΔMdn* = −0.67, *z* = −2.60, *p* = 0.010).

Despite the nonexistence of statistically significant differences between groups regarding social competence reported by parents, and externalizing behaviors, within group analysis revealed a significant increase of social competence from baseline to 12 weeks in the RG (*ΔMdn* = 0.14, *z* = −2.57, *p* = 0.010), and in externalizing behaviors in the WCG (*ΔMdn* = −0.10, *z* = −2.60, *p* = 0.011).

Regarding the acute effects in salivary cortisol levels, as shown in Table 4, there was statistical differences at 24th pre-session between LPPG and RG, in which the LPPG had more 0.09 μg/dL than RG. Results show no significant difference between salivary cortisol concentration at 1st and 24th pre-session and post-session. Despite this, in the 24th session, the salivary cortisol concentration significantly decreased from pre-session to post-session in the LPPG (*ΔMdn* = −0.10, *z* = −2.60, *p* = 0.010).

**Table 4:** Scores on cortisol at pre-and post-session.

Cortisol	Group	Pre-session *Mdn* (IQR)	Post-session *Mdn* (IQR)	Kruskal–Wallis *H* value of *p*
Session 1	LPPG	0.23 (0.11)	0.16 (0.04)	0.87
	RG	0.15 (0.11)	0.11 (0.11)	
	CG	0.08 (0.13)	0.11 (0.05)	
Session 24	LPPG	0.21 (0.17)^a^	0.15 (0.09)*	0.09
	RG	0.12 (0.07)^a^	0.09 (0.05)	
	CG	0.11 (0.14)	0.13 (0.14)	

Note: Data are expressed as median and interquartile range (IQR). ^a^*p* < 0.05 comparison between groups at baseline. *p < 0.05 changes within the group using the Wilcoxon test. LPPG, loose parts play group; LPP, loose parts play group; CG, combined group.

## 4. Discussion

The present study aimed to examine the effects of three body-oriented interventions on preschoolers’ social–emotional competence. Both Loose parts play and Relaxation programs increased preschoolers’ positive emotion expression and several within-groups changes were found for the Loose parts play, the Relaxation and the Combined programs, suggesting the effectiveness of the three programs to promote emotion discrimination and identification, and self-regulation. From baseline to post-intervention, both Loose parts play and Relaxation programs improved children’s positive emotion expression but only children from the relaxation group improved their social competence. Throughout the intervention period, children from the control group decreased their conflict resolution abilities and increased externalizing problems.

All schools that were contacted to implement the programs accepted to participate in the study. Such high level of acceptance might be related to the recognition of the importance of social–emotional development by early-childhood education professionals. Also, preschool teachers seem to acknowledge the critical role of outdoor time for preschoolers’ health and well-being. Moreover, the low time commitment (two biweekly 30-min sessions) might have facilitated the acceptance of the program.

The mean attendance rate of children to the programs was 82%, which is considered a good rate comparing to other body-oriented intervention programs (Goldstein and Lerner, 2017). Children were informed that they were free to engage in other activities, during the session time. However, this never happened and children always choose to participate in the sessions, showing highly motivated to participate. Hence, the level of attendance reveals school absence. A high level of motivation may be related to the fact that sessions enabled children to engage in active and playful activities that they were not used to engaging. Moreover, sessions were implemented in the outdoors and therefore were an opportunity to be outside, and to experience the freedom of movement and expression. Also, especially in the Loose parts play program and in the Combined program, children had the chance to play with original objects/materials that there were not used. Finally, the warm presence of the psychomotor therapist and the preschool teacher might have been important to the high level of motivation.

In what concerns to the effects of the three body-oriented programs, our findings showed that both Loose parts play and Relaxation programs effectively increased positive emotion expression. Although studies with older school aged children had already shown the positive effects of a relaxation intervention on the expression of positive emotions (Alba, 2013; Lindsay et al., 2018), this is the first study to extend these findings to younger children. In fact, relaxation states have been related to positive emotions. In particular, when children are calm and more relaxed, they can experience and express more positive emotions (Amutio et al., 2015; Fredrickson et al., 2017). It is important to note the active and playful dimension of the relaxation intervention implemented. Such physical activity and playfulness (Chang et al., 2013) might have also benefited positive emotion expression.

In what concerns to the effects of the Loose parts play program on children’s expression of positive emotions, the perceived freedom throughout the session, might have facilitated the experience and expression of positive emotional states. Although two other studies had already shown that play-based intervention programs effectively promote children’s positive emotion expression (Moore and Russ, 2008; Rao and Gibson, 2021), these studies did not focus on loose parts play (i.e., pretend play; Moore and Russ, 2008) or combined play with other body-oriented approaches (i.e., loose parts play and mindfulness; Lee et al., 2020). Altogether these findings reinforce the potential of loose parts play to promote children’s positive emotion expression.

Considering the positive effects of both body-oriented approaches (Loose parts play and Relaxation) on children’s positive emotion expression, the lack of effects of the Combined intervention program was not expected. Such finding may be explained by the reduced time available for both approaches (15 min for loose parts play, 10 min for relaxation), considering that in a previous study focused on a combined program, the loose parts play part had an hour duration (Lee et al., 2020).

Our findings suggest that relaxation might be the best body-oriented approach to promoting social competence. The relaxation program involved a progression in terms of the activities’ social level. The first sessions comprised individual relaxation activities, then sessions progressed to activities in pairs, and later to group activities. Such progression, embedded in a playful atmosphere, might have benefited the development of social skills. These findings are in line with a previous study that showed the effectiveness of a 12-week mindfulness intervention on preschoolers’ social competence (Flook et al., 2015).

Our findings also showed that all the intervention programs effectively improved emotion discrimination and identification, which goes in line with a previous study showing that relaxation (Richard et al., 2019) enhances emotion understanding. However, only the Relaxation program effectively increased children’s emotion recognition. Contrary to the discrimination and identification tasks, emotion recognition was assessed through parents’ perspectives on children’s abilities to recognize their parents’ emotional expressions. In the Relaxation program children were first guided to observe and become aware of their own body sensations and expressions, and later to observe others’ bodies and expressions, and to establish the relationship between bodily and emotional states. Such focus and progression might have been important for children to become attentive and aware of others’ emotion expressions. Nevertheless, the lack of effects of the combined program on emotion recognition suggests that these gains require time and orientation, since the combined program only involved a 10-min relaxation practice.

Although a recent systematic review showed contradictory findings for the benefits of body-oriented interventions in preschoolers’ self-regulation (Dias Rodrigues et al., 2022b), our findings show that, after the 12-week intervention period, the three intervention groups showed an improved self-regulation measured by the HTKS, in comparison to the control group who showed decreased self-regulation. These findings are in line with previous studies (Chinekesh et al., 2014; Duman and Ozkur, 2019; Loukatari et al., 2019) and reinforce the idea that integrating specific outdoor body-oriented moments in the early-childhood curriculum are critical for preschoolers’ self-regulation development.

Concerning self-regulation measured by salivary cortisol levels, there were no significant differences between groups neither before nor after the intervention period. Also, cortisol levels measured throughout the 1st session, did not vary in any of the programs. However, it is important to note the significant decrease in salivary cortisol levels during the 24th session of the Loose parts play program. Such a decrease suggests that children who had more opportunities to freely play with loose parts, improved their ability to reduce physiological arousal, unlike the other intervention groups. It is important to note that contrary to Relaxation and Combined sessions, Loose part play sessions were child-directed. Hence, the experienced freedom during loose parts play intervention might be particularly beneficial for children’s self-regulation.

## 5. Limitations and future research

This study has some limitations. Participants’ allocation to the groups was not randomized. Besides, the small sample size influences the observation of positive effects. Henceforth, future studies should consider replicating of the present study with a random allocation, and a higher sample size. Besides, it is important to run a follow-up evaluation in order to examine the long-term effects of the intervention programs.

## 6. Implications and conclusion

This study examined the effects of three body-oriented intervention programs on preschoolers’ social–emotional competence. Both Loose parts play and Relaxation programs increased preschoolers’ positive emotion expression and several within-groups improvements were found for the loose parts play, relaxation and combined programs, suggesting the potential effectiveness to promote emotion discrimination and identification, and self-regulation. This study reinforces the importance of integrating outdoor and body-oriented approaches into the preschool curriculum for children’s social–emotional development.

## Data availability statement

The raw data supporting the conclusions of this article will be made available by the authors, without undue reservation.

## Ethics statement

The studies involving human participants were reviewed and approved by University of Évora Ethics Committee. Written informed consent to participate in this study was provided by the participants’ legal guardian/next of kin.

## Author contributions

ADR, GV, JM, and CP contributed to the design and conception of this study and cautiously designed the intervention program. GV, JM, and CP supervised the implementation of the study. ADR collected the data, which was analyzed by ADR, GV, and JM. EL performed the saliva analyses. ADR wrote the first draft of the manuscript. ADR, GV, JM, EL, DG, and CP contributed to manuscript revision, read, and approved the submitted version. All authors contributed to the article and approved the submitted version.

## Funding

This work was funded by national funds through the Foundation for Science and Technology, under the project UIDP/04923/2020.

## Conflict of interest

The authors declare that the research was conducted in the absence of any commercial or financial relationships that could be construed as a potential conflict of interest.

## Publisher’s note

All claims expressed in this article are solely those of the authors and do not necessarily represent those of their affiliated organizations, or those of the publisher, the editors and the reviewers. Any product that may be evaluated in this article, or claim that may be made by its manufacturer, is not guaranteed or endorsed by the publisher.
